# Children in foster care with symptoms of reactive attachment disorder: feasibility randomised controlled trial of a modified video-feedback parenting intervention

**DOI:** 10.1192/bjo.2022.538

**Published:** 2022-07-18

**Authors:** Paula Oliveira, Lydia Barge, Eloise Stevens, Sarah Byford, James Shearer, Ruan Spies, Julie Comyn, Kirsty Langley, Paul Ramchandani, Barry Wright, Matt Woolgar, Eilis Kennedy, Stephen Scott, Jane Barlow, Danya Glaser, Rob Senior, Peter Fonagy, Pasco Fearon

**Affiliations:** Division of Psychology and Language Sciences, University College London, UK; and Anna Freud National Centre for Children and Families, UK; Institute of Psychiatry, Psychology and Neuroscience, King's College London, UK; School of Psychosocial Health, North-West University, South Africa; Division of Psychology and Language Sciences, University College London, UK; Faculty of Education, University of Cambridge, UK; Hull York Medical School, University of York, UK; South London and Maudsley NHS Trust, UK; and Institute of Psychiatry, Psychology and Neuroscience, King's College London, UK; Research and Development Unit, Tavistock and Portman NHS Foundation Trust, UK; Department of Social Policy and Intervention, University of Oxford, UK; Great Ormond Street Hospital for Children, UK; and Division of Psychology and Language Sciences, University College London, UK; Division of Psychology and Language Sciences, University College London, UK; Anna Freud National Centre for Children and Families, UK; and Centre for Family Research, University of Cambridge, UK

**Keywords:** Attachment disorder, foster care, parenting intervention, feasibility, randomised controlled trial

## Abstract

**Background:**

Looked-after children are at risk of suboptimal attachment patterns and reactive attachment disorder (RAD). However, access to interventions varies widely, and there are no evidence-based interventions for RAD.

**Aims:**

To modify an existing parenting intervention for children with RAD in the UK foster care setting, and test the feasibility of conducting a randomised controlled trial (RCT) of the modified intervention.

**Method:**

The intervention was modified with expert input and tested on a case series. A feasibility and pilot RCT compared the new intervention with usual care. Foster carers and children in their care aged ≤6 years were recruited across nine local authorities, with 1:1 allocation and blind post-treatment assessments. The modified intervention was delivered in-home by trained mental health professionals over 4–6 months. Children were assessed for RAD symptoms, attachment quality and emotional/behavioural difficulties, and foster carers were assessed for sensitivity and stress.

**Results:**

Minimal changes to the intervention programme were necessary, and focused on improving its suitability for the UK foster care context. Recruitment was challenging, and remained below target despite modifications to the protocol and the inclusion of additional sites. Thirty families were recruited to the RCT; 15 were allocated to each group. Most other feasibility outcomes were favourable, particularly high numbers of data and treatment completeness. The revised intervention was positively received by practitioners and foster carers.

**Conclusions:**

A large-scale trial may be feasible, but only if recruitment barriers can be overcome. Dedicated resources to support recruitment within local authorities and wider inclusion criteria are recommended.

Children in care are at greatly increased risk of experiencing mental health problems related to their adverse experiences, which often include maltreatment, and separation from and frequent changes in caregivers.^1^ These experiences have been clearly demonstrated to have significant consequences in the development of attachments.^2^ ‘Attachment problems’ among children in care, including reactive attachment disorder (RAD), disinhibited social engagement disorder (DSED) and disorganised attachment, are important targets for intervention.^3^ RAD and DSED are disorders^4^ observed almost exclusively among children who have experienced extreme neglect and/or repeated changes in caregivers.^5^ Disorganised attachment is a developmental vulnerability factor that may arise in the context of high-risk caregiving environments, including maltreatment and highly insensitive/atypical parenting, and that increases the likelihood of future adjustment difficulties.^5,6^

Remarkably few interventions exist with proven efficacy for intervening on behalf of, or preventing, poor outcomes for looked-after children,^7^ and existing interventions to improve emotional/behavioural difficulties and attachment quality have not been tested rigorously, particularly in the context of foster care.^8–10^ In addition, no study has yet tested whether any intervention is effective in the treatment of attachment disorders or the symptoms within this population,^11^ which is a significant omission, given their likely prevalence in the foster care population.^12^ Critically, the number of children in care has been rising steadily in the UK in recent years, with 80 850 children currently in care, and the majority of them in foster placements.^13^ There is, therefore, an urgent need to develop effective and cost-effective interventions and make them available for this large group of highly vulnerable children.

This study was commissioned to develop and pilot an intervention programme for children in foster care who show symptoms of RAD. This is a complex enterprise because despite clinical concern about RAD in this population, there is great uncertainty about its aetiology and prevalence in foster care. Given this limited evidence, it is unclear what is the most appropriate approach to intervention. Nevertheless, given that a central feature of RAD is the absence of consistent attachment behaviour (such as proximity-seeking), those factors known to promote secure attachments are arguably the most promising candidates. There is strong evidence that sensitive and responsive caregiving is a primary driver of secure parent–child attachment,^14^ and is therefore a logical target for increasing a child's attachment-related proximity-seeking and secure base behaviour toward a caregiver. Furthermore, there is good evidence that sensitive-responsive caregiving is linked to a range of wider positive developmental outcomes.^15^ Intervening to maximise sensitive responsiveness for children in foster care therefore has the potential to improve RAD symptoms specifically, and to promote their broader socioemotional development and well-being.

## Selection of intervention

Interventions promoting caregiver sensitivity have the best evidence of effectiveness for reducing attachment problems among children in care. Video-feedback methods have been recommended specifically because of their good efficacy and cost-effectiveness profiles.^3,11^

The Video-Feedback Intervention to Promote Positive Parenting and Sensitive Discipline (VIPP-SD), in particular, represents a promising brief and cost-effective treatment for children in foster care.^16,17^ The VIPP-SD is a rigorously tested, sensitivity-focused video-feedback intervention, with demonstrated improvements in parental sensitivity to the child's attachment cues,^17^ and has been used in a broad range of contexts with young children. The programme was recently modified for the Dutch foster care context,^18^ with the aim of addressing the sometimes complex attachment behaviours and needs of foster children. However, no studies have tested the effectiveness of VIPP-SD for increasing attachment security or reducing attachment problems in a UK healthcare setting for children in foster care.

## Study aims

The first aim of this study was to adapt the VIPP-SD programme to meet the specific needs of children with attachment difficulties captured by the term RAD, in the UK foster care system. The second was to test the modified intervention on a small case series. The third aim was to test the feasibility of a future full-scale trial of effectiveness and cost-effectiveness, by conducting a pilot randomised controlled trial (RCT) of the modified intervention and evaluating a number of feasibility parameters, including recruitment flow and the performance of screening tools for RAD, and the candidate primary outcome measure, including estimates of measure variances, intervention acceptability, documentation of standard care and the feasibility of a health economic evaluation.

## Method

### Design

A mixed-methods study was conducted with several interlinked phases. First, we adapted the treatment manual with expert input, trained intervenors to deliver the programme, and then tested it on a small case series. In the main phase of the study, we conducted a pilot RCT of the modified intervention. Children and their carers taking part in the RCT were randomly assigned to the modified VIPP-SD intervention (henceforth designated VIPP-Foster Care (VIPP-FC)) plus care as usual (CAU) or to CAU alone. This study was conducted in out-patient National Health Service (NHS) child and adolescent mental health services (CAMHS) and partner local authority social services departments in England, including urban and rural/semirural areas.

Results from a scoping study in which key stakeholders from local authorities and CAMHS were interviewed to optimise the study protocol were published elsewhere,^19^ and the complete findings from qualitative interviews with foster carers that received the new intervention, to further assess its acceptability, will be published separately.

The study protocol can be accessed from an online repository.^20^ This study was registered with the ISRCTN registry (identifier ISRCTN18374094) on 22 May 2017, and the Central Portfolio Management System (identifier 34889).

### Ethics and governance

The authors assert that all procedures contributing to this work comply with the ethical standards of the relevant national and institutional committees on human experimentation and with the Helsinki Declaration of 1975, as revised in 2008. All procedures involving human patients were approved by the London-Harrow Research Ethics Committee (approval number 17/LO/0987). The complex consent process for recruiting for the case series and the RCT was designed based on consultation with experts and local authorities, and was carried out in two stages: first, for the screening stage (which involved no direct contact with children), local authorities sent an opt-out letter to birth parents, and, subsequently, foster carers also had to provide a signed consent form when returning the screening questionnaires; second, for taking part in the randomisation/intervention stage, both foster carers and all those with parental responsibility for the child were required to sign a consent form (with the exception of children on a full care order where the local authority deemed it not in the child's best interests to seek birth parent consent). Active refusal of consent or decision to withdraw the child's participation by a parent was always respected, regardless of the child's legal status. Verbal assent was obtained from children aged 4 years and above.

Structured oversight of the project was undertaken by a Trial Steering Committee and a Data Monitoring and Ethics Committee. The data were collected and processed in accordance with the General Data Protection Regulation and the Data Protection Act of 2018.

### Participants

Participants in the case series and in the pilot RCT were foster or kinship carers with children presenting RAD symptoms, aged between 11 months and 6 years. The target sample size for the feasibility RCT was 40, with 20 allocated to each arm of the trial. The age range was selected based on the appropriateness of the intervention and of the outcome measures, including taking account of the uncertainty of reliably identifying RAD symptoms in older children. An additional inclusion criterion required that the placement was planned to continue for at least 4 months, to allow sufficient time to complete the intervention. The only exclusion criteria, beyond carer's insufficient cognitive or language skills, were that the carer was already engaged in a similar parenting intervention, or severe intellectual disability of the child. It is important to note that during the last period of the trial, we implemented an amendment to allow inclusion of children that did not necessarily present RAD symptoms. This was implemented to address recruitment challenges to the necessary throughput of cases, and also in recognition of the fact that all children in foster care are likely to be at heightened risk for RAD and a range of other attachment difficulties, and therefore potentially able to benefit from VIPP-FC.

Table 1 shows the key demographic data collected at the initial screening and at entry to the RCT.

**Table 1:** Participant demographic characteristics

	Participants	
Characteristic	Screening (*n* = 96)	Randomised controlled trial (*n* = 30)
Child's gender, *n* (%)		
Male	58 (60)	17 (57)
Female	38 (40)	13 (43)
Child's age (months), mean (s.d.)	47.92 (24.01)	47.37 (21.20)
Child's legal status, *n* (%)		
Full care order	57 (60)	24 (80)
Interim care order	22 (23)	4 (13)
Special guardianship order	11 (11)	2 (7)
Voluntary care (Section 20)	3 (3)	
Unknown	3 (3)	
Total time in foster care (months), mean (s.d.)	17.32 (14.06)	18.13 (15.03)
Carer's gender, female, *n* (%)	86 (90)	29 (97)
Carer type		
Foster carer	81–74%	24 (80%)
Family and friends carer	19–26%	6 (20%)
Child's ethnicity^a^, *n* (%)		
White British	35 (52)	14 (47)
Caribbean	6 (9)	2 (7)
White/Caribbean mixed	5 (8)	3 (10)
Other ethnicities	21 (31)	11 (36)
Child's first placement,^a^ *n* (%)		
Yes	35 (52)	15 (50)
No	32 (48)	15 (50)
Carer's age^a^ (years), mean (s.d.)	51.86 (8.32)	49.87 (9.66)
Carer's support,^a^ *n* (%)		
Shared carer	42 (63)	20 (67)
Single carer	25 (37)	10 (33)
Carer's ethnicity^a^, *n* (%)		
White British	50 (75)	22 (73)
Caribbean	8 (12)	3 (10)
Other ethnicities	9 (13)	5 (17)
Carer's experience (years), mean (s.d.)	6.59 (6.35)	5.97 (5.91)

a. These data were available only for the subsample completing the Disturbances of Attachment Interview visit, *n* = 67.

### Recruitment and randomisation

Recruitment was implemented in partnership with local authorities children's services. Initial contact with participants was established via a large community screening, in which local authorities sent an invitation to complete screening questionnaires to potentially eligible foster carers (based on child age and type of placement). Consenting carers who returned the screening questionnaires to the research team and were potentially eligible (based on the eligibility criteria described under ‘Participants’) were invited to a more detailed face-to-face interview assessment to confirm eligibility and gather data about the performance of the screening measures. Where eligibility was confirmed and all necessary consents obtained, foster carers were invited to participate in the RCT. Baseline and post-treatment assessments took place either in the local authority premises or partner services, to reduce travel time for participants.

Participants were individually randomised in a 1:1 ratio to one of the two treatment arms: the VIPP-FC plus CAU or CAU only. Randomisation with minimisation was employed by an unblinded member of the trial coordination team with the program SiMin for Windows,^21^ with minimisation factors age (≤4 *v*. >4 years), gender and site. All data handling and communication regarding unblinded information was undertaken by the trial coordinating team. All outcome assessments were done by research assistants blind to treatment allocation. By definition, foster families were not blind to the treatment arm they had been randomised to.

### Measures

#### Screening

There is currently no pre-existing established tool to screen for RAD symptoms in clinical trials, and we therefore used two questionnaires jointly to optimise sensitivity for detecting relevant cases: the Development and Well-Being Assessment (DAWBA) section on ‘Relationships with Adults’,^22^ and the Attachment Screening Assessment (ASA)^23^ (see Tables 2 and 3).

**Table 2:** Descriptive statistics for the trial outcome measures, at baseline

	CAU arm score, *n* = 15					VIPP-FC arm score, *n* = 15				
Outcome measure	*n*	Mean	s.d.	Minimum	Maximum	*n*	Mean	s.d.	Minimum	Maximum
DAI–RAD	15	2.40	1.72	0	5	15	2.73	2.02	0	6
DAI–DSED	15	2.60	2.26	0	6	15	2.33	2.32	0	6
ASA–RAD	14	23.86	6.32	12	34	13	23.54	8.50	13	35
ASA–DSED	14	9.71	5.22	5	20	12	8.67	5.76	5	21
ASA–secure	14	42.29	6.51	31	55	13	43.31	9.39	21	52
ASA–avoidant	14	22.86	5.71	12	31	13	21.62	8.72	11	35
ASA–resistant	13	12.46	3.99	6	18	12	12.50	4.15	6	20
ASA–disorganised	13	18.08	4.41	11	24	12	18.92	7.49	10	36
ASA–PCP	13	11.62	4.66	5	21	12	10.58	4.42	5	18
ASA–PCC	12	12.17	4.80	5	21	12	12.25	5.24	7	24
DAWBA–RAD	15	3.13	1.41	1	5	14	2.36	2.21	0	8
DAWBA–DSED	15	5.47	3.34	1	11	14	5.14	4.11	0	12
CBCL–internalising	13	15.23	11.01	3	41	15	14.67	12.75	0	39
CBCL–externalising	13	20.08	14.05	3	41	15	21.20	13.87	0	41
CBCL–total problems	13	53.69	33.09	12	96	15	55.67	39.33	3	113
PSES–total	13	21.92	2.66	17	25	15	21.80	2.78	16	25
PSI–parental distress	13	47.24	7.25	32	60	13	52.54	5.84	42	60
PSI–parent-child	12	47.00	7.27	32	57	14	46.43	8.54	33	58
PSI–difficult child	12	41.67	10.65	26	60	14	44.14	10.60	27	60
PSI–total stress	11	135.36	21.19	98	167	13	145.00	21.89	106	177
NICHD–sensitivity	15	3.26	0.42	2.31	3.88	15	3.24	0.36	2.56	3.94
	*n*	Category = *n* (%)				*n*	Category = *n* (%)			
DAWBA–any disorder	12	≥70% likelihood = 2 (17)				12	≥70% likelihood = 3 (25)			
		~50% likelihood = 3 (25)					~50% likelihood = 2 (17)			
		≤15% likelihood = 7 (58)					≤15% likelihood = 7 (58)			
SDQ–any disorder	12	Probable = 6 (50)				12	Probable = 5 (42)			
		Unlikely/possible = 6 (50)					Unlikely/possible = 7 (58)			
SSP pattern	15	Secure = 8 (53)				15	Secure = 11 (73)			
		Avoidant = 4 (27)					Avoidant = 3 (20)			
		Ambivalent = 0 (0)					Ambivalent = 0 (0)			
		Controlling/disorganised = 1 (7)					Controlling/disorganised = 0 (0)			
		Insecure-other = 2 (13)					Insecure-other = 1 (7)			

CAU, care as usual; VIPP-FC, Video-feedback Intervention to Promote Positive Parenting and Sensitive Discipline–Foster Care; DAI, Disturbances of Attachment Interview; RAD, reactive attachment disorder/inhibited subscale; DSED, disinhibited social engagement disorder/disinhibited subscale; ASA, Attachment Screening Assessment; PCP, pattern controlling–punitive; PCC, pattern controlling–caregiving; DAWBA, Development and Well-Being Assessment; CBCL, Child Behaviour Check-List; PSES, Parenting Self Efficacy Scale; PSI, Parenting Stress Index; PSI–parent-child, Parenting Stress Index parent–child dysfunctional interaction; NICHD-Sensitivity, National Institute of Child Health and Human Development Scales–sensitivity composite; SDQ, Strengths and Difficulties Questionnaire; SSP, Strange Situation Procedure.

**Table 3:** Descriptive statistics for the trial outcome measures, at follow-up

	CAU arm score, *n* = 15					VIPP-FC arm score, *n* = 15				
Outcome measure	*n*	Mean	s.d.	Minimum	Maximum	*n*	Mean	s.d.	Minimum	Maximum
DAI–RAD	15	1.87	1.64	0	5	13	2.23	2.28	0	6
DAI–DSED	15	2.87	2.23	0	6	13	1.61	2.26	0	6
ASA–RAD	12	23.58	6.92	13	32	10	23.00	6.70	15	34
ASA–DSED	12	8.58	3.90	5	16	11	7.73	4.38	5	17
ASA–secure	12	42.75	6.02	33	52	1	42.70	6.75	35	51
ASA–avoidant	12	22.33	5.96	12	31	1	21.27	6.44	11	33
ASA–resistant	12	12.50	3.87	7	20	1	14.27	4.94	8	24
ASA–disorganised	12	16.75	4.73	10	24	1	18.27	6.31	10	29
ASA–PCP	12	11.58	3.63	5	15	1	11.00	4.84	5	20
ASA–PCC	12	10.83	3.88	6	17	1	11.90	3.41	6	18
DAWBA–RAD	13	1.92	1.89	0	5	10	2.10	1.66	0	5
DAWBA–DSED	13	5.08	3.57	0	11	10	4.00	4.85	0	13
CBCL–internalising	13	14.85	10.36	2	31	11	14.82	13.01	0	35
CBCL–externalising	13	20.46	11.11	6	39	11	21.27	15.26	0	39
CBCL–total problems	13	50.92	29.82	15	98	11	57.18	40.93	0	118
PSES–total	14	22.36	2.50	18	25	11	22.27	3.50	15	25
PSI–parental distress	14	46.00	6.78	32	59	11	51.27	7.39	36	60
PSI–parent-child	14	45.50	7.30	32	56	10	46.70	7.50	37	57
PSI–difficult child	14	42.50	8.81	29	56	11	45.72	11.15	28	60
PSI–total stress	14	134.00	20.41	104	169	10	144.90	23.96	104	174
NICHD–sensitivity	14	3.12	.37	2.44	3.69	12	3.15	0.47	2.31	3.88
	*n*	Category: *n* (%)				*n*	Category: *n* (%)			
DAWBA–any disorder	12	≥70% likelihood = 2 (17)				12	≥70% likelihood = 3 (25)			
		~50% likelihood = 3 (25)					~50% likelihood = 2 (17)			
		≤15% likelihood = 7 (58)					≤15% likelihood = 7 (58)			
SDQ–any disorder	12	Probable = 6 (50)				12	Probable = 5 (42)			
		Unlikely/possible = 6 (50)					Unlikely/possible = 7 (58)			
SSP pattern	14	Secure = 9 (65)				12	Secure = 7 (58)			
		Avoidant = 2 (14)					Avoidant = 2 (17)			
		Ambivalent = 0 (0)					Ambivalent = 2 (17)			
		Controlling/disorganised = 1 (7)					Controlling/disorganised = 0 (0)			
		Insecure-other = 2 (14)					Insecure-other = 1 (8)			

CAU, care as usual; VIPP-FC, Video-feedback Intervention to Promote Positive Parenting and Sensitive Discipline–Foster Care; DAI, Disturbances of Attachment Interview; RAD, reactive attachment disorder/inhibited subscale; DSED, disinhibited social engagement disorder/disinhibited subscale; ASA, Attachment Screening Assessment; PCP, pattern controlling–punitive; PCC, pattern controlling–caregiving; DAWBA, Development and Well-Being Assessment; CBCL, Child Behaviour Check-List; PSES, Parenting Self Efficacy Scale; PSI, Parenting Stress Index; PSI–parent-child, Parenting Stress Index parent–child dysfunctional interaction; NICHD-Sensitivity, National Institute of Child Health and Human Development Scales–sensitivity composite; SDQ, Strengths and Difficulties Questionnaire; SSP, Strange Situation Procedure.

#### Primary outcome

The Disturbances of Attachment Interview (DAI)^24^ is a semi-structured interview to evaluate the presence of signs of disordered attachment. The first five items of the interview address signs of emotionally withdrawn/inhibited attachment disturbance (i.e. RAD symptoms; scores can range from 0 to 10; intraclass correlation coefficient = 0.99; *n* = 22), and the next three items address signs of indiscriminate behaviour (i.e. DSED symptoms; scores can range from 0 to 6; intraclass correlation coefficient = 0.99; *n* = 22). There is sound evidence for the validity of this instrument.^25,26^ Raters were blinded to treatment allocation. Note that the DAI was used to assess research diagnostic criteria for purposes of this study and not to establish a clinical diagnosis, which would entail a clinician-led comprehensive assessment.

#### Secondary outcomes

Child attachment classifications were assessed in the Strange Situation Procedure,^27,28^ which was adapted for administration in a standard room with a secure live streaming camera (to avoid the need for participants to travel to a laboratory with a one-way mirror). Co-occurring difficulties were reported by the carer on the DAWBA^22^ and the Child Behaviour Checklist.^29^ Measures of parenting included the carer's sensitivity on the National Institute of Child Health and Human Development scales^30^ during carer–child interaction, and ratings on the Parenting Stress Index^31^ and Parental Self-Efficacy Scale.^32^ Coders of observational measures (Strange Situation Procedure and National Institute of Child Health and Human Development scales) were blinded to timepoint, treatment allocation and placement characteristics.

#### Economic measure

The Child and Adolescent Service Use Schedule (CA-SUS)^33^ was modified for the current study, based on versions used with similar populations,^34,35^ to measure the use of all health, social care and education-based services and to support the description of usual care.

### Existing versions of the intervention

The VIPP-SD is a brief home-based attachment and parenting intervention, focused on improving carer's sensitivity to the child. Accredited practitioners visit families at home for seven 90 min sessions, where they video-record carer–child interactions that are then used as a basis for themed discussions with the carer in the next session. Themes are consistent across families, although the video feedback is personalised.

The adaptation of the VIPP-SD to the foster care context in the Netherlands focused on specifically addressing the attachment difficulties often shown by children in foster care, such as being aware that they may present attachment signals in a very subtle, highly distorted or absent way, or how to gently and sensitively support a child's need for physical contact and comfort in a safe way.

### Data analysis

Feasibility parameters (means, proportions and variance estimates) were assessed with 95% confidence intervals. Summary and descriptive statistics were used for quantitative outcome measures, but no inferential statistics were used for most analyses because of the small sample size. Qualitative methods and process records were used to address questions regarding foster carer perceptions of treatment acceptability and delivery. The modified CA-SUS was tested for comprehensiveness (missing items) and non-redundancy of services, and databases were searched to identify approaches to the measurement of health utilities for cost–utility analysis in this young age group.

## Results

### Intervention adaptation

The first phase of the study aimed to refine the VIPP-FC programme to the UK foster care context and specifically for children with difficulties in the realm of RAD. The adaptation was based on input from a working group of experienced clinicians, and learning acquired during the case series. The expert group recommended minor changes to the intervention protocol, and endorsed the content and approach of the intervention for use with foster children and their carers.

Some modifications involved changes to the manual itself, including improved translation of messages or games to the British context, and tailoring the language to the specific circumstances of the child and their placement. The duration was also compressed to six sessions, with a shorter gap to the final (booster) session, to minimise the chances of disruption by a change in placement. Other adjustments were related to the broader context for delivery of the intervention, such as the introduction of a goal-based outcome measure and a closing letter to share with the social worker, to aid embedding of the intervention in routine UK mental health services and the network of professionals working with children in care.

### Training, supervision and fidelity

We trained VIPP-SD intervenors in our modified VIPP-FC programme and provided accredited supervision. Intervenors were clinicians from NHS specialist looked-after children mental health services or other appropriately qualified practitioners. Fidelity to the programme was ensured through supervision of three visits per case, completion of logbooks by intervenors, and audio-recording of at least one feedback session per case.

### Testing the intervention and procedures in a case series

A small case series, based in one site, to road-test the study procedures and the modified VIPP-FC programme, reinforced the suitability of the intervention and the assessments, but highlighted key challenges to recruitment that were important to address for the pilot RCT. These were a smaller recruitment pool than initial estimates, resulting from the local authority deeming many cases inappropriate to approach, and a lower than expected return rate of screening questionnaires (only 60% compared with 80% expected). The process observations highlighted the critical importance of social worker involvement and engagement in the project, as well as administrative support from the local authority to encourage carers to return the questionnaires and engage with the research team.

In response to these challenges, key changes implemented for the RCT were inclusion of additional study sites to increase the pool of participants, mechanisms to facilitate return of screening questionnaires (e.g. online form; social workers to obtain verbal consent from carers to be directly contacted by the research team) and identification of an administrator or ‘study champion’ at each site to facilitate communication and engagement between the research team and social workers and carers.

### Conducting a pilot RCT of the modified intervention

The RCT involved eight NHS trusts and nine linked local authorities, and took place across four large regions in England: Greater London (North, South, East and West), Hertfordshire, Peterborough and Yorkshire. As noted previously, a substantial number of local authorities was needed to ensure adequate recruitment and to capture important variability in organisational contexts that may be important to understand when preparing a future larger-scale clinical trial.

### Participant flow

Recruitment was implemented in stages, initially by screening participants with two questionnaires that were mailed out by the local authority and returned by the participant to the research team by post. Carers were then visited to establish, using a validated and rigorous investigator-led diagnostic schedule (i.e. the DAI), whether or not children showed evidence of RAD symptoms. Although the RAD symptoms eligibility criterion was removed toward the end of the study, we continued to use all outcome measures to track impact on RAD symptoms, as well as other clinical domains. Consenting eligible families were invited to the RCT.

**Fig. 1:**
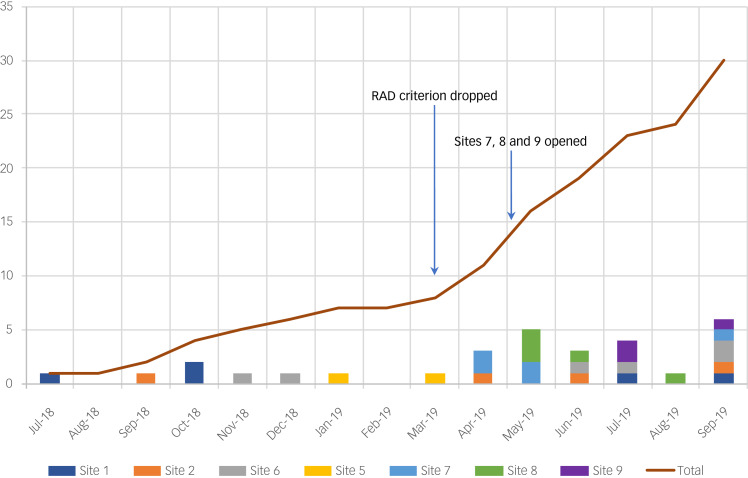
Recruitment rate over time, with number of participants recruited on the *y*-axis. RAD, reactive attachment disorder.

Of the nine sites that initially agreed to take part, we were able to recruit successfully from seven. Recruitment was extended from the originally planned period of 12 months to 17 months. The timeline and key events against progress on recruitment are illustrated in Fig. 1.

**Fig. 2:**
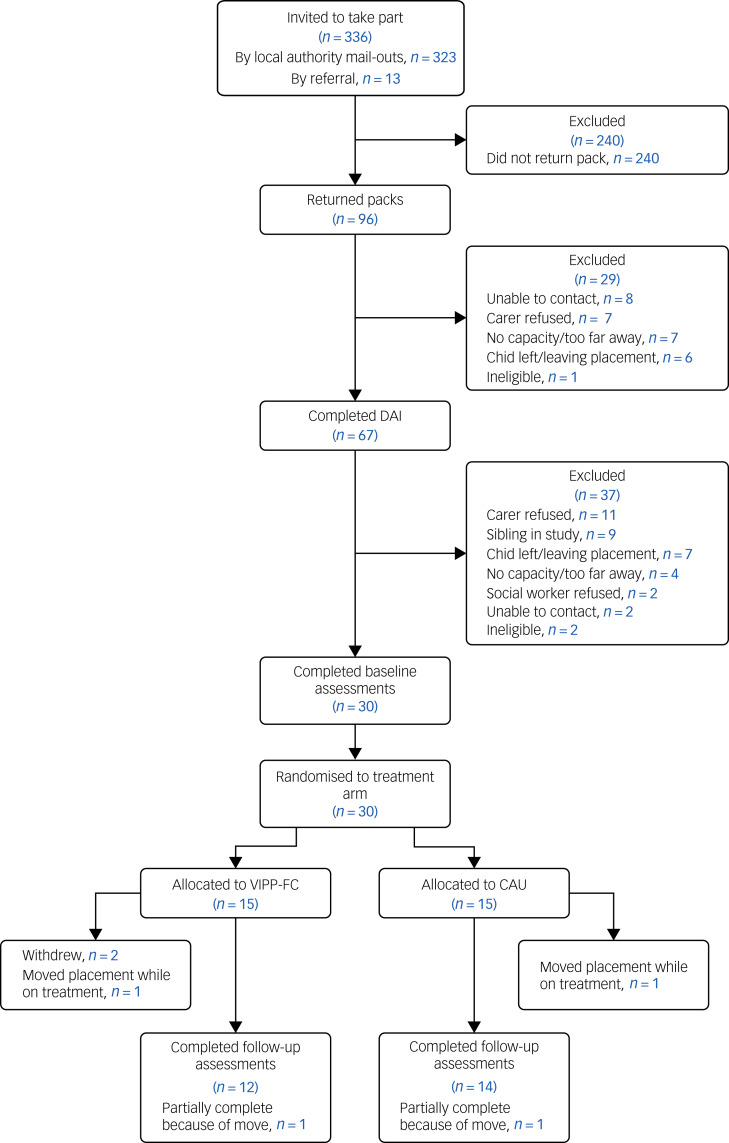
CONSORT diagram for the VIPP-FC pilot trial. CAU, care as usual; DAI, Disturbances of Attachment Interview; VIPP-FC, Video-Feedback Intervention to Promote Positive Parenting in Foster Care.

Despite implementing a range of small adjustments to the protocol and inclusion of additional sites, we encountered significant challenges to recruitment in the pilot RCT. The most significant barriers occurred during the initial stages of (indirect) contact with foster carers. The overall number of mailed-out questionnaires (*n* = 336) was lower than the target of 500, largely because of significant numbers of children registered with the local authorities being considered inappropriate or ineligible for the study (e.g. very short placements, placed out of borough). The overall response rate to the screening questionnaires was 29% (95% CI 24–34%), which is substantially below the target of 80% and below the rate achieved in the case series (60%). It is important to note that the return rate varied substantially per site, from 3 to 63%. The overall response and retention rates are shown via a CONSORT diagram in Fig. 2.

Recruitment flow increased substantially in the last period of the study, as can be seen in Fig. 1. The improved recruitment rates largely coincided with the removal of the RAD criterion, the addition of three new sites with administrative capacity available to support the research, and our success in one site in obtaining an honorary contract for a member of the research team to work within children's services to directly support recruitment. In this final phase, the rate of recruitment was four cases per month, but these were primarily from just four sites. By the end of the recruitment period, we recruited and randomised 30 cases to the trial, which was ten cases below the original target.

### Evaluation of feasibility outcomes

#### Feasibility of identifying appropriate screening instruments

Non-parametric ROC curve analysis indicated that the two screening questionnaires performed well against the DAI (i.e. assuming the DAI as gold standard) in terms of sensitivity and specificity for case detection. The estimated area under the curve was 0.84 (95% CI 0.74–0.92) and 0.83 (95% CI 0.71–0.91) for the DAWBA and ASA, respectively.

#### Feasibility of identifying sufficient numbers of appropriate cases with screening instruments

Results are presented in Table 4, and show that despite low return rates, the questionnaires performed well.

**Table 4:** Feasibility of identifying sufficient numbers of appropriate cases with screening instruments

Feasibility parameter	Proportion	95% CI
Screening questionnaires returned from the total sent out	0.29	0.24–0.34
Positively screened cases (above upper 66th percentile)^a^ agreeing to full assessment on the DAI	0.73	0.60–0.86
Positively screened cases (above upper 66th percentile) on either screening questionnaire that scored ≥3 on the DAI (i.e. true positive rate)	0.86	0.71–1.0
Cases with a DAI RAD score of ≥3, but not meeting the screening threshold on either questionnaire (i.e. false negative rate)	0.09	0.00–0.21

DAI, Disturbances of Attachment Interview; RAD, Reactive Attachment Disorder.

a. The 66th percentile was the planned threshold to consider positive cases in the screening questionnaires.

#### Feasibility of recruiting/consenting foster families with children aged <7 years presenting with RAD symptoms

Results are presented in Table 5, and show that once carers were in direct contact with the research team, recruitment was less challenging (see also Fig. 2).

**Table 5:** Feasibility of recruiting foster families with children aged <7 years presenting with reactive attachment disorder symptoms

Feasibility parameter	Proportion	95% CI
Cases consenting to enter the pilot RCT from those meeting entry criteria (screening questionnaires + DAI) – when there was a RAD criterion for eligibility	0.56	0.41–0.71
Cases consenting to enter the pilot RCT from those meeting entry criteria (screening questionnaires + DAI) – if RAD criterion is ignored for eligibility	0.45	0.33–0.57
Conversion rate into trial from total cases invited to take part overall (before screening)	0.9	0.6–0.12

RCT, randomised controlled trial; DAI, Disturbances of Attachment Interview; RAD, reactive attachment disorder.

#### Feasibility of randomising to VIPP-FC or CAU, and intervention throughput and acceptability

No eligible participants refused randomisation. Post-treatment outcome assessments were always undertaken after treatment completion, and therefore the duration of follow-up across treatment arms was balanced by yoking cases in each arm. On average, the actual period between baseline to follow-up was 185 days (s.d. = 67) in the VIPP-FC arm and 160 days (s.d. = 56) in the CAU arm. Of the 15 participants randomised to VIPP-FC, four did not proceed to receive it: two because of lack of clinical capacity in the study site, another two dropped out from the study because of social worker concerns about competing interventions that had been initiated since initial contact. All other participants completed 100% of the planned sessions. Acceptability was further assessed via qualitative interviews with foster carers who received the intervention. Foster carers’ experiences of VIPP-FC were very positive, endorsing the valuable contribution of the research and a range of benefits of the intervention, from enhanced skills to improved relationship with their child. The complete findings from these interviews will be published separately.

#### Feasibility and acceptability of baseline and outcome assessments

Assessment measures and procedures were in general seen as appropriate and there were few missing data points. Missing outcome assessments post-treatment were because of the two participants dropping out after randomisation mentioned above. In two other cases, children moved to a new placement, precluding some of the outcome assessments. See Tables 2 and 3 for data completeness. All assessments were acceptable, although some challenges were experienced by some carers who had difficulties accessing the online-based DAWBA. No problems were encountered with our adaptation of the Strange Situation Procedure laboratory procedure to a portable system.

### Candidate primary outcomes

#### Establish the approximate prevalence of elevated RAD symptoms

The results of the DAI indicated an approximate prevalence of significant RAD symptoms in this sample of 33% (22 out of 67 assessed) (95% CI 22–44%). This proportion is based on cases scoring ≥3 on the total RAD score, which indicates clinically significant difficulties in the areas assessed by any of the five items.

#### Identify the most appropriate primary outcome

Both questionnaire measures of RAD demonstrated good convergence and sensitivity against the research-diagnostic interview. Nevertheless, consideration of acceptability supported the use of the DAI, as foster carers preferred the interview format to the paper questionnaires. Although the sample size was too small to complete formal estimates of the sensitivity of the measures for detecting change, it was notable that the DAWBA (RAD subscale) and the DAI showed an overall trend for reductions from pre- to post-treatment (across both arms of the trial, including all participants with follow-up data; DAWBA *t* = 2.35, *P* = 0.03; DAI *t* = 1.95, *P* = 0.06), whereas the ASA did not (*t* = −0.07, *P* = 0.95)). On balance, we judge that the DAI should be the preferred tool for reliably assessing RAD symptoms for a trial of this nature (as long as appropriate training is provided to interviewers/raters), and the DAWBA scale may be most suitable for screening.

#### Obtain initial estimates of the variance of key outcome measures.

Tables 2 and 3 summarise baseline and post-treatment descriptive statistics for the main outcome variables. To obtain an approximate estimate of the standard deviation of the RAD symptom measures for future trial planning, we relied on the screening data, for which the sample size was largest, including data collected for the case series and RCT. For the DAWBA-RAD subscale, the variance was 5.0, and for the ASA-RAD subscale, it was 52.3 (upper 80% confidence limits of 6.07 and 64.8, respectively) (based on 101 screening cases). For the DAI-RAD subscale, the variance was 4.16 (upper 80% confidence limit of 5.2) (based on 73 interviews). We do not report tests of group difference because these would not be meaningful, given the small sample size.

#### Obtain estimates of the variance of secondary outcome measures

Descriptive statistics for the main outcome measures are presented for future trial planning (see Tables 2 and 3). Participating children experienced high levels of emotional and behavioural difficulties. Results from the CBCL indicated average problems were in borderline clinical range. Similarly, the Strengths and Difficulties Questionnaire and the DAWBA indicated likely significant difficulties for approximately half the children. On the other hand, the majority of children were classified as secure in their relationship with their carers. Lastly, foster carers experienced high levels of parenting stress, with average scores on all subscales above the 95th percentile.

### Document CAU

VIPP-FC was compared with CAU, as there is no pre-existing integrated care pathway for children in foster care and no routine assessment of or intervention for attachment difficulties. We used the CA-SUS to systematically describe and quantify the services received by children and carers in the comparator arm of the pilot RCT; our aim was not to determine whether or not they related specifically to RAD, but to describe which services participants were using and how frequently. Findings indicated that children receive a diverse set of services related to their development and well-being. The services that are offered include universal services related to being in care (e.g. social worker visits), services for children identified as requiring additional support (e.g. carer consultation or one-to-one support at school) or for any other reasons. See Table 6 for a list of the reported services.

**Table 6:** Services reported in the description of usual care

Items	% Completed	Categories selected
Accommodation	100	Formal foster care (local authority) Formal foster care (agency) Formal kinship care
Education	100	Mainstream school Preschool or nursery Other: portage, special needs school, early risers/breakfast club, small support group, booster classes
Hospital services	100	In-patient Out-patient Accident and emergency
Community services	100	General practitioner Nurse at the surgery Community nurse Community paediatrician Child's social worker Supervising social worker Adoption social worker Family support worker Mental health services worker Counsellor Clinical psychologist Psychiatrist Community psychiatric nurse Educational psychologist Play therapist Art/drama/music therapist Speech and language therapist Physiotherapist Dietician Occupational therapist Audiologist
Medication	100	(Various acute or long-term prescribed medications)

### Test feasibility of conducting health economic evaluation

Feedback from research assessors and levels of completeness suggested that the CA-SUS was acceptable to both interviewers and interviewees. Minor adjustments were made to some wording, a few redundant items were removed (e.g. residential mother/baby facility) and a few missing items were added (e.g. portage).

In terms of utility measures, proxy versions for children aged <7 years are available for two measures (the Child Health Utility 9-Dimensions (CHU9D) and the youth version of the EQ-5D-3L (EQ-5D-Y)), but we found no evidence to support their validity or reliability. In addition, the EQ-5D-Y proxy measure is for use with children aged 4–7 years and, although no minimum is stated for the CHU9D, it is possible that neither measure is suitable for children aged 0–3 years. It is therefore not currently possible to recommend any specific measures for inclusion in a future trial of VIPP-FC, and thus we recommend that the primary economic evaluation should be a cost-effectiveness analysis, using the primary clinical outcome measure. We further recommend that a future trial of VIPP-FC may be a good opportunity to compare the construct validity and sensitivity to change of the proxy versions of existing instruments by exploring the relationship of these measures with appropriate clinical outcomes.

## Discussion

There is very limited evidence regarding effective programmes for addressing RAD symptoms and/or insecure or disorganised attachment of children in foster care, and improving their emotional and behaviour outcomes. At the same time, many researchers working in this field have noted the serious challenges to outcome research inherent in the systems around children in care, which explains in part the relatively scant and methodologically weak evidence base.^36,37^ This feasibility study focused on adapting the VIPP-SD parenting intervention to the UK foster care context and the needs of children in care with symptoms of RAD. We then sought to conduct a feasibility study to assess the extent to which a full-scale RCT might be possible.

VIPP-SD is a well-evidenced intervention informed by both attachment and social learning models. Sessions are delivered in-home by a trained intervenor, who frames their feedback to the carer into a coherent attachment-informed message, drawing attention to the child's cues and communications, the carer's sensitive response and the positive effect this has on the child. In this study, we adapted the programme to the context of children in foster care in the UK with RAD symptoms or at high risk of presenting RAD symptoms. The expert manual development group implemented a series of minor changes to the intervention programme that focused on improving its suitability for the UK foster care context and endorsed the content and approach of the intervention as highly appropriate for use with foster carers and foster children.

A small case series to road-test the intervention programme and trial procedures revealed many crucial challenges to recruitment that needed resolving before proceeding to the pilot RCT. Despite implementing a range of adjustments to the protocol to address these challenges, we continued to experience difficulties with recruitment in the RCT. The most significant barriers were encountered during the initial stages of contact with foster carers: the significant proportion of cases that local authorities deemed inappropriate or ineligible for the study, and the low return rate of screening questionnaires. We were unable to recruit the target sample size of 40 after numerous initiatives were introduced to increase recruitment, including – in stages – allowing online screening, engaging additional sites and, finally, allowing all children in foster care to enter the study even if RAD diagnostic criteria were not met. By the end of the study, we were able to recruit three-quarters of our target sample size into the RCT, with 15 families allocated to each treatment arm.

Notwithstanding the difficulties with recruitment, many other features of the pilot trial design worked well. The screening tools performed well in identifying potentially eligible participants, demonstrating good convergence and sensitivity against the research diagnostic interview. We achieved good outcome data completeness and all the participants who received the treatment completed all sessions. Recipients of the intervention expressed very positive views about their experience during qualitative interviews, including enhanced skills and improved relationship with their child, and found the programme acceptable and helpful.

Feasibility work relating to the health economic evaluation indicated that the CA-SUS is acceptable and worked well with this population. Nevertheless, there are currently no appropriate measures to calculate quality-adjusted life years for use in cost–utility analysis; therefore we recommend that in a future trial of VIPP-FC, the primary economic evaluation should be reliant on cost-effectiveness analysis, focusing on the primary clinical measure of outcome.

The implications of this study were clear: the intervention itself and the research protocol for evaluating its impact were robust and implementable to a high degree of rigour, but recruitment problems persisted. We were able to identify key ingredients and strategies to facilitate recruitment, but these were only possible to implement in a limited way during this feasibility study. Of critical importance is the social worker's involvement and engagement in the research, which is difficult to achieve for different reasons including their heavy caseloads, but that can be partially addressed by identifying individuals within the local authority to link with the research team and provide effective administrative support. This message was echoed by the stakeholders interviews.^19^ In a future trial, recruitment should be supported by adequate resourcing within local authorities, increasing capacity within the research team to be present in each recruitment site to a greater degree to promote engagement of social work teams, and allowing direct contact between the research team and all eligible foster carers. An additional strategy would be to more actively engage families on a special guardianship order, which were difficult to access in the current study, largely because they tended to be managed by different teams in local authorities.

Furthermore, findings from this study and the refinements that were introduced to the intervention manual suggest that a future trial should not select on the basis of RAD diagnosis, but instead address the efficacy of VIPP-FC for children in foster care in general – potentially including RAD as an outcome measure. An avenue for future work is to explore the implementation of a trial of VIPP-FC as part of universal foster carer training, which would largely remove most barriers to recruitment while enhancing foster carers skills and providing more immediate and direct benefit to children than more pedagogical approaches commonly used in the foster care sector.

To conclude, collaborators and stakeholders were clear that progressing this work is important. The fundamental barrier to progression to a full-scale trial was recruitment, but challenges are surmountable by implementing modifications to recruitment strategies. A future trial would need significant dedicated resourcing for recruitment and greater or more seamless integration with the participating local authorities. Embedding a trial within universal foster carer training/continuing professional development and/or widening the scope to include special guardians and children adopted should also be considered. Recognising the need to find alternative solutions for challenges of this nature, there is growing support by funders (e.g. National Institute for Health and Care Research studies within a trial) to find ways to improve the processes that allow successful completion of studies.

## Data Availability

All data requests should be submitted to the corresponding author, P.O., for consideration.
